# Dental Hygienists’ Practice in Perioperative Oral Care Management According to the Japanese Dental Hygienists Survey 2019

**DOI:** 10.3390/ijerph18010114

**Published:** 2020-12-26

**Authors:** Yoshiaki Nomura, Yuki Ohara, Yuko Yamamoto, Ayako Okada, Noriyasu Hosoya, Nobuhiro Hanada, Noriko Takei

**Affiliations:** 1 Department of Translational Research, Tsurumi University School of Dental Medicine, Yokohama 230-8501, Japan; hanada-n@tsurumi-u.ac.jp; 2 Japanese Dental Hygienists’ Association, Tokyo 169-0071, Japan; yohara@tmig.or.jp (Y.O.); nori@pm-ms.tepm.jp (N.T.); 3 Research Team for Promoting Independence and Mental Health, Tokyo Metropolitan Institute of Gerontology, Tokyo 173-0015, Japan; 4 Department of Endodontology, Tsurumi University School of Dental Medicine, Yokohama 230-8501, Japan; yamamoto-y@tsurumi-u.ac.jp (Y.Y.); hosoya-n@tsurumi-u.ac.jp (N.H.); 5 Department of Operative Dentistry, Tsurumi University School of Dental Medicine, Yokohama 230-8501, Japan; okada-a@tsurumi-u.ac.jp

**Keywords:** dental hygienist, perioperative oral care management, oral care practice

## Abstract

Perioperative oral care management is effective in the prevention of postoperative complications, and dental hygienists play an important role. The aim of this study was to elucidate the practice and substantial role of dental hygienists in perioperative oral care management. The Japan Dental Hygienists Association conducted a survey of the employment status of Japanese dental hygienists in 2019. Questionnaires were distributed to all 16,722 members, and 8932 were returned (collection rate: 53.4%). A total of 3560 dental hygienists were working at dental clinics and 1450 (38.2%) were performing perioperative oral care management. More than 90% of them implemented conventional oral care practice. Less than half of the dental hygienists implemented treatment concerning oral functions. Only 9.9% of dental hygienists recognized shortened hospital stay as an effect of perioperative oral care management. Dental hygienists who implemented both nutritional instruction and training of swallowing function or mouth rinsing instructions recognized the shortened hospital stay effect. Overall implementation of perioperative oral care management led to shortened hospital stay. Implementation of perioperative oral care management required knowledge and skills related to systemic health conditions. A stepwise educational program is necessary for perioperative oral care management to become commonplace for dental hygienists.

## 1. Introduction

Postoperative complications lead to serious problems, including prolonged hospital stay and increased morbidity, mortality and cost [1,2,3]. Perioperative care plays an important role in the prevention of postoperative complications. Perioperative oral care management is especially effective [4] in the reduction of the incidence of postoperative respiratory infections, [4,5,6,7,8,9], surgical site infections [10], length of hospital stay [5,10,11,12] and mortality [7,11].

Perioperative oral care management includes conventional oral hygiene practice: infected teeth extraction, removal of dental plaque and calculus, cleaning of the tongue coating and dentures and instructions regarding self-care. Moisture retention of the oral cavity, training of swallowing function and nutritional instruction in accordance with the patient’s oral conditions are also involved [13]. Dental hygienists play an important role in these procedures.

In Japan, the national medical insurance system covers a wide range of medical and dental treatment. Perioperative oral care management has been introduced into this insurance system. According to the analysis of the nationwide administrative claim database in Japan for this insurance system, 509,179 patients underwent resection of head and neck, esophageal, gastric, colorectal, lung or liver cancer between May 2012 and December 2015. Of these patients, 81,632 (16.0%) received preoperative oral care from a dentist [6]. According to the Japanese national survey, the number of hospitals was 8355 [14], and the number of dentists who work at hospitals was 3162 (2018) [15]. The number of dentists who can supply perioperative dental care is not sufficient. To reinforce perioperative oral care management, two systems are now available. Patients consult a dentist at a dental clinic for oral hygiene procedures and instructions before hospitalization for surgery. Dentists and dental hygienists who regularly work at dental clinics are employed by hospitals as part-time attendees/on a part-time basis to carry out perioperative oral care management. In 2019, there were 68,477 dental clinics that supported perioperative oral care management.

The Japan Dental Hygienists Association conducts a survey of dental hygienists every five years [13,16]. In the recent survey, items concerning perioperative dental care were included in the questionnaire. There are several important roles of perioperative oral care management: improving oral masticatory functions, reducing the amount of oral bacteria, removing infection sources and preventing accidents during hospitalization. To achieve all of these tasks in a limited period is not easy. The skills of dental hygienists are varied [17]. Therefore, it is important to investigate the practice conducted by dental hygienists in perioperative oral care management in order to disseminate the information for perioperative oral care management. To improve patient satisfaction, a medical team approach has been recommended to utilize the specialty of medical staff. Dental hygienists need to enroll in specific medical teams for effective oral care management implementation [18,19]. In this study, practices of dental hygienists in perioperative oral care management were analyzed in combination with team participation and the self-assessed effect of perioperative oral care management.

Perioperative oral care management contains many elements: implementation rate, procedures, effects and recognition of dental hygienists and their interactions. However, information on the present situation is scarce. The aim of this study was to elucidate the actual procedures and roles of dental hygienists in perioperative oral care management.

## 2. Materials and Methods

### 2.1. Survey Method

The Japan Dental Hygienists Association has been conducting surveys of the employment status of Japanese dental hygienists every five years since 1981 [13,16]. Questionnaires were distributed to all members of the Japan Dental Hygienists Association on 16 October 2019 by post, including a stamped addressed envelope for recovery. The survey execution date was set as 31 December 2019. The questionnaires returned up to 30 November were used for the analysis. One of the limitations of this study was this sampling frame. The study population was limited to the members of the Japan Dental Hygienists Association.

### 2.2. Questionnaire

The questionnaire used in this study consisted of 101 items concerning demographic factors, employment situation, working procedures, willingness to work, etc. The items concerning demographics were common to all the dental hygienists. Specific items were related to their current or most recent workplace: dental clinic or hospital, government, nursing home or school.

The results for the dental hygienists working at dental clinics or hospitals who answered “yes” for the implementation of perioperative oral care management were analyzed. Three major items concerning perioperative oral care management were analyzed in this study. These three items were procedures of perioperative oral care management (13 kinds of procedures, multiple choice), self-assessed effects of perioperative oral care management (13 kind of symptoms or statuses of the patients, multiple choice) and participation in a medical care team (10 kinds of medical care team, multiple choice). These items were all dichotomous. The contents of each item are shown in Table 1 in combination with the results of descriptive statistics.

The questionnaire in Japanese is available from the Japan Dental Hygienists Association [20].

### 2.3. Statistical Analysis

A three-parameter logistic model with item response theory (IRT) analysis was applied to calculate item discrimination, difficulty and guessing [13,21,22,23]. Item response and item information curves were graphically illustrated. The analyses were carried out by R software v 3.50 with the LTR and irtoys packages using the following formula:

(1) $$P_{i}\left(\theta \right)=\frac{\left(1-c_{i}\right)}{1+e^{-Da_{i}\left(\theta -b_{i}\right)}}$$

where *a_i_*: discrimination, *b_i_*: difficulty and *c_i_*: guessing.

Logistic regression analysis was performed to find the practice of perioperative oral care management concerned with each self-assessed effects of perioperative oral care management.

A cross-tabulation was performed on the self-assessed effect of perioperative oral care management and participation in medical care teams on the procedures of perioperative oral care management. Correspondence analysis was performed with this cross-tabulation. The results were illustrated graphically as biplots [24,25]. Decision analysis was carried out using classification and regression trees (CARTs) to determine the factors associated with the self-assessed effect of shortened hospital stay [26,27]. SPSS Statistics Ver 24.0 (IBM, Tokyo, Japan) was used for these analyses.

## 3. Results

### 3.1. Demographic Characteristics of the Dental Hygienists Implementing Perioperative Oral Care Management

The questionnaire was distributed to 16,722 members of the Japan Dental Hygienist Association, and 8932 were returned (collection rate: 53.4%). Among the 3560 dental hygienists working at dental clinics, 1450 (38.2%) performed perioperative oral care management. The participants included 1448 women and 2 men. Age and experience as a dental hygienist were not statistically significantly linked to performing perioperative dental care (implemented: age: 45.67 ± 11.93, experience: 19.64 ± 11.26 years; not implemented: age: 45.10 ± 12.10, experience 18.73 ± 11.30 years).

### 3.2. Descriptive Statistics of the Items Investigated in This Study

The number of dental hygienists practicing the treatments, self-assessed recognition of treatment effects and rate of participation in medical teams are summarized in Table 1.

### 3.3. Item Response Analysis of the Items Concerning Perioperative Oral Management

**Figure 1:**
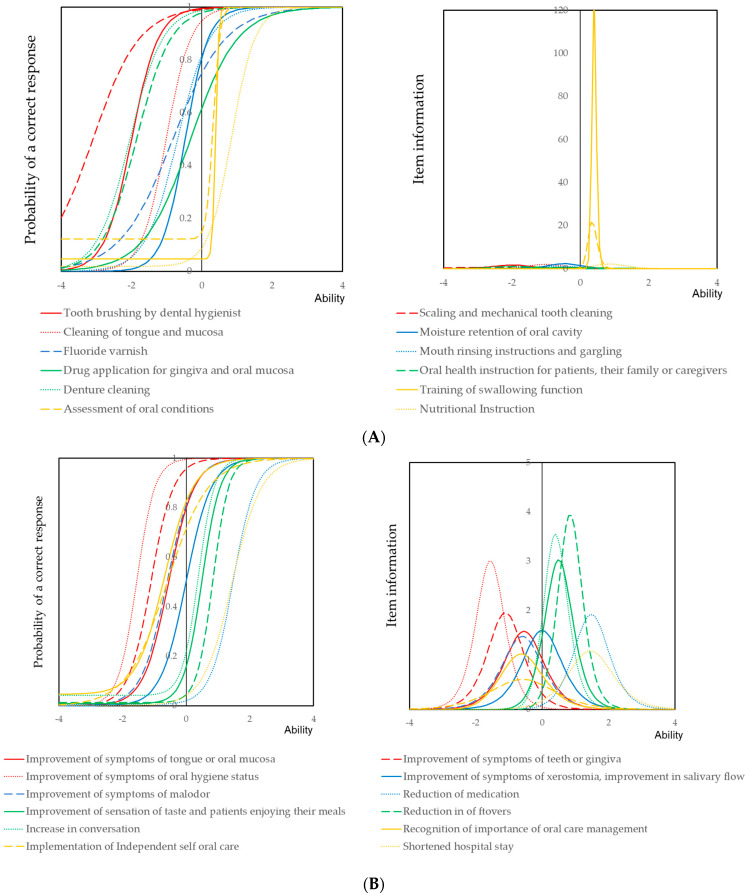

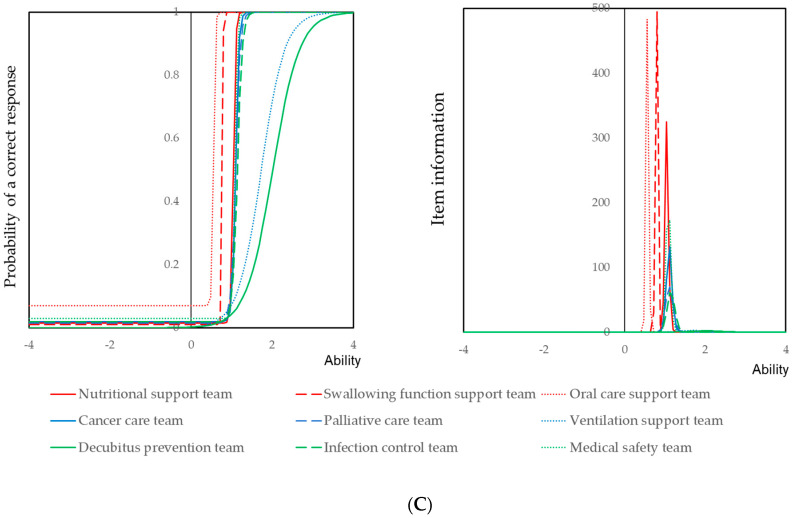
Item response curve and item information curves for the items concerning perioperative oral management. (**A**) Treatments of perioperative oral care management, (**B**) self-assessed perioperative oral care management and (**C**) participation in a medical team. Ability, the scale of the X-axis, represents the weighted score of the total rate of implementation. An item response curve or item information curve with a backward direction indicates that the item was frequently implemented. In contrast, an item response curve with a forward direction indicates that the item was rarely implemented. Steep item response curves and high item information curves indicate that when these items were implemented, other items were easily implemented.

Figure 1 shows the item response curves and item information curves for the treatments of perioperative oral care management (A), self-assessed perioperative oral care management (B) and participation in a medical team (C). Hand brushing by a dental hygienist was easy to implement. Nutritional instructions were hard to implement. Eating and swallowing instructions had the highest item information. Implementation of eating and swallowing instructions had the least item information, given the multiplicity of the skills of dental hygienists involved in perioperative oral management. Symptoms of tongue or oral mucosa and oral hygiene status were easy to improve. Reduction of medication and shortened hospital stay were hard to achieve. The oral care team had the highest level of participation, while the prevention of decubitus and ventilation support teams had the lowest. The level of participation in an oral care team was 199 (13.8%). Models constructed by item response theory are presented in Table S1.

### 3.4. Self-Assessed Effects of Perioperative Oral Care Management

Self-assessed effects of oral of perioperative oral care management were analyzed by logistic regression analysis (Table S2). Practices of dental hygienists were used for independent variables. Cleaning, oral hygiene instructions and drug application were effective for the improvement of oral hygiene and symptoms in the oral cavity. Nutritional instruction by dental hygienists had various effects.

### 3.5. Correlations between Treatments of Perioperative Oral Care Management, Self-Assessed Perioperative Oral Care Management and Participation in a Medical Team

**Figure 2:**
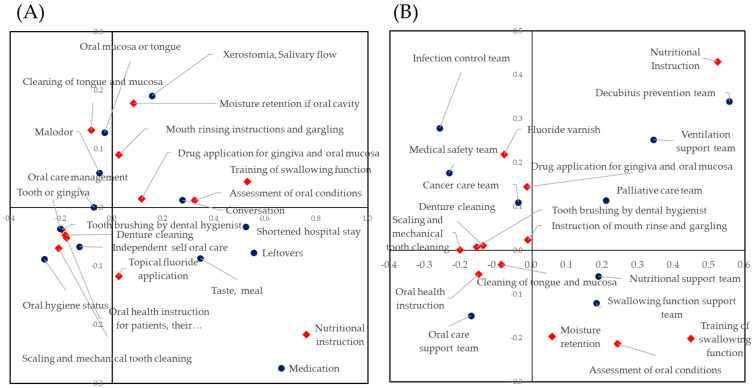
Biplots of treatments and self-assessed effects (**A**) and treatments and participation in a medical team (**B**). Red plots correspond to the treatment of perioperative oral care management. Navy plots correspond to the self-assessed effect of perioperative oral care management. Closely located plots were highly coincident.

Cross-tabulations of treatments and self-assessed effects (A) and treatments and participation in medical team (B) are shown in Table S3. To visualize the interrelations of these items’ three categories, correspondence analysis was carried out based on the cross-tabulations. Figure 2 shows the biplots of treatments and self-assessed effects (A) and treatments and participation in a medical team (B). As shown in Figure 2A, items of oral hygiene instruction, hand brushing and mechanical tooth cleaning were closely located. Items concerning the effects of perioperative oral care management surrounded the items concerning treatment. These items were the improvement of oral hygiene, symptoms of teeth and gums, independence of oral hygiene and understanding of the importance of oral hygiene. Tongue and mucosal cleaning was located near the improvement of symptoms of tongue and oral mucosa. The humidity retention of the oral cavity was located near the improvement of oral dryness. Nutritional instruction by dental hygienists was located far from effects and other treatments.

For treatments and participation in a medical team, the team’s approaches to cancer treatments, oral care and medical safety included treatments concerning cleaning and oral hygiene instructions (Figure 2B).

### 3.6. Treatments and Treatment Effects Led to Shortened Hospital Stay

**Figure 3:**
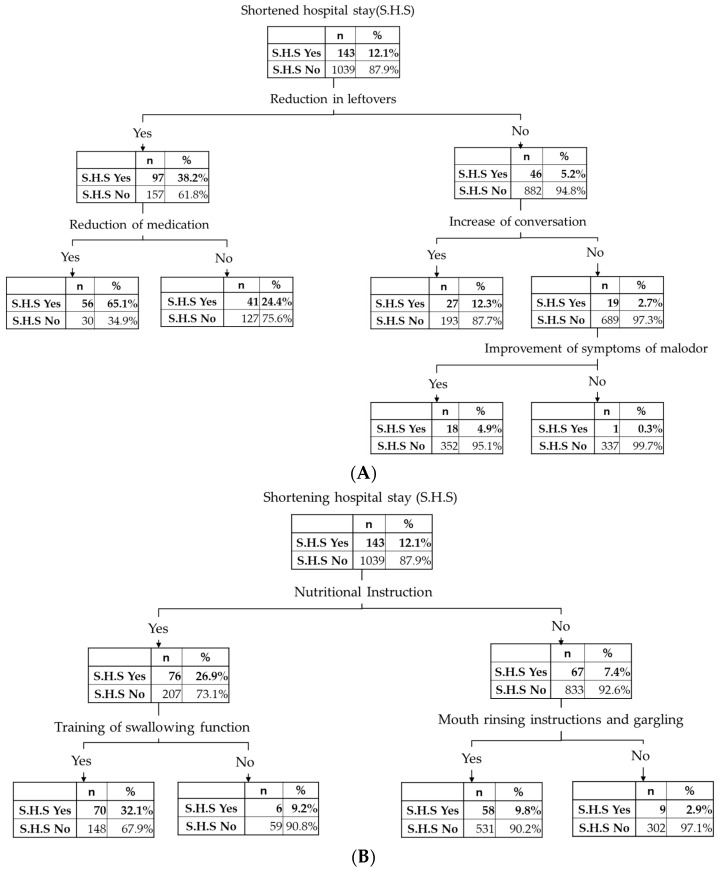
Decision trees used to determine processes and effects leading to a shortened hospital stay: (**A**) treatment that led to a shortened hospital stay; (**B**) self-assessed effects of perioperative oral care management that led to a shortened hospital stay. Subjects who did not answer for the item of shortened hospital stay were excluded from the analysis.

Among the self-assessed treatment effects, shortened hospital stay is an important and major outcome. To determine the factors affecting the shortened hospital stay, a decision analysis was carried out. For the treatments, more than half of the dental hygienists who recognized the shortened hospital stay effect carried out nutritional instructions. Among the dental hygienists who carried out both nutritional instructions and training of eating and swallowing functions, 70 dental hygienists recognized shortened hospital stay. Among the dental hygienists who did not carry out nutritional instructions and recognized the shortened hospital stay effect, 58 dental hygienists carried out mouth washing instructions (Figure 3A).

Other treatment effects may also lead to a shortened hospital stay. A decision analysis was carried out again by using self-assessed treatment effects. Among the 143 dental hygienists who recognized a shortened hospital stay, 97 also recognized a reduction of leftovers (Figure 3B).

## 4. Discussion

In this study, data from 1450 dental hygienists who worked at dental clinics and implemented perioperative oral care management were investigated. Among the 3560 dental hygienists working at dental clinics, 1450 (38.2%) performed perioperative oral care management. More than 90% of them implemented conventional oral care practice: tooth cleaning, denture cleaning and oral health instructions. The item response curves of these items, shown in Figure 1A, were shifted in a backward direction. These conventional oral health procedures reduce the risk of respiratory infections [28,29,30,31,32,33,34,35]. The implementation of oral moisture management, instruction of mouth rinsing and application of drugs, including topical fluoride application, was less than 70%. Less than half of the dental hygienists implemented treatment concerning oral functions. Item information curves of these items had a forward direction. Item information of these items were very high. These results indicate that dental hygienists who implemented oral function treatment conducted most of the perioperative oral care management items listed in Table 1. Stepwise training and education may be necessary for the dental hygienists who need to implement perioperative oral care management.

For the self-assessed effect of perioperative oral care management, item response curves of the improvement of symptoms concerning oral hygiene showed a backward direction (Figure 1B). This indicated that many dental hygienists recognized that the symptoms concerning oral hygiene were improved by their interventions. In contrast, dental hygienists who recognized the effect of reduction of medication and shortening of hospital stay were limited. Item response curves of these items had a forward direction. As shown in Figure 2A, the plots of conventional oral care practice and self-assessed effects of the improvement of symptoms concerning oral hygiene were aggregated around the starting point. Plots concerning the practice and self-assessed effects of oral functions were located around conventional oral care practice. Self-assessed effects of a shortened hospital stay, improvement of taste, reduction of leftovers and reduction of medication were located around nutritional instruction. Poor oral health was strongly associated with malnutrition [36]. Patients with malnutrition exhibited worsening conditions during hospitalization [37]. Dental hygienists recognized nutritional instruction may affect systemic health status. Intervention of oral functional training is known to be effective in improving swallowing function [12,38,39]. This leads to the reduction of leftovers and improvement of nutritional status [40,41]. As seen in the results of logistic regression analysis shown in Table S2, nutritional instruction was statistically significant for most of the self-assessed effects of perioperative oral care management. This result and the location of the item response curve of nutritional instruction indicated that dental hygienists who implemented nutritional instruction recognized that perioperative oral care management was effective for the improvement of most of the symptoms investigated in this study. The plots for nutritional instruction and participation in a nutritional support team were not closely located in the biplot shown in Figure 2B. A dietitian may participate in the nutritional support team. Dental hygienists practicing nutritional instruction indicated that nutritional instruction in accordance with improving masticatory and swallowing functions may be carried out [36,38]. These dental hygienists may recognize the overall effect of preparative oral management.

Shortened hospital stay is one of the major outcomes of the effect of perioperative oral care management. Shortening hospital stay is important because the Japanese insurance system introduced a flat system for many types of surgery. This has led to cost reduction by the hospital management. The item response curve of shortened hospital stay had a forward direction (Figure 2B). This result indicated that dental hygienists recognized most of the effects listed in Table 1 when they recognized a shortened hospital stay. As seen in the results of logistic regression analysis shown in Table S2, the odds ratio of nutritional instruction was highest. The item response curve of nutritional instruction had a forward direction, and these results indicated that the overall implementation of perioperative oral care management leads to shortened hospital stay. According to the results of decision analysis, only 143 dental hygienists recognized the effect of a shortened hospital stay. Among them, more than half of the dental hygienists implemented nutritional instruction (75/143, 52.4%). Most of the dental hygienists who recognized the shortened hospital stay effect implemented both nutritional instruction and training of swallowing function or mouth rinsing instructions (128/143, 89.5%). As shown in Figure 3B, within the self-assessed effect, dental hygienists who recognized a shortened hospital stay recognized the effect on the reduction of leftovers, increase in conversation or improvement of malodor (142/143, 99.3%).

The effect of oral care management was not applicable for all hospitalized patents. A study showed that for the patients who underwent esophagectomy, oral care management did not have a significant effect on mean hospital stay and mortality rate [42]. Another study showed that oral care management was ineffective in hospitals with an incidence of postoperative pneumonia of more than 20% [43]. The ability of dental hygienists to recognize effects may differ according to the situation of the hospital or patients. This is one of the limitations of this study. The situation of dental hygienists and the conditions of the patients were not included in the study.

In Japan, the number of dental hygienists is not sufficient. Based on 2018 figures, there were 104,908 dentists, and among them, 59,482 were owners of private dental clinics [15]. The number of currently working dental hygienists was 132,629 [44]. There were 68,500 private dental clinics in Japan in 2020 [14]. Therefore, there are many dental hygienists that do not work in a clinic. The number of dentists, dental hygienists or dental clinics that can provide preoperative oral care management has been limited. Therefore, infrastructure reform is indispensable in providing a sufficient supply of dental health services for current and future demands.

In summary, less than 40% of dental hygienists worked on the perioperative oral care management at dental clinics. Many of them implemented conventional oral care practice. Dental hygienists recognized several effects of perioperative oral care management in accordance with the increase of the implementation of treatments that need knowledge and skills related to systemic health conditions.

## 5. Conclusions

Dental hygienists recognized the effects of oral care on improvement of overall health status. A stepwise educational program is necessary for perioperative oral care management to become commonplace for dental hygienists.

## Figures and Tables

**Table 1:** Descriptive statistics of the items investigated in this study.

	Items	*n*	%
Practice of perioperative oral care management	Tooth brushing by dental hygienist	1371	94.6%
	Scaling and mechanical tooth cleaning	1405	96.9%
	Cleaning of tongue and mucosa	1157	79.8%
	Moisture retention if oral cavity	937	64.6%
	Topical fluoride application	987	68.1%
	Mouth rinsing instructions and gargling	1004	69.2%
	Drug application for gingiva and oral mucosa	826	57.0%
	Oral health instruction for patients, their family or caregivers	1319	91.0%
	Denture cleaning	1352	93.2%
	Training of swallowing function	446	30.8%
	Assessment of oral conditions	632	43.6%
	Nutritional instruction	317	21.9%
Self-assessed effect of perioperative oral care	Improvement of symptoms of tongue or oral mucosa	834	57.5%
	Improvement of symptoms of teeth or gingiva	1033	71.2%
	Improvement of symptoms of oral hygiene status	1165	80.3%
	Improvement of symptoms of xerostomia, improvement in salivary flow	626	43.2%
	Improvement of symptoms of malodor	859	59.2%
	Reduction of medication	129	8.9%
	Improvement of sensation of taste and patients enjoying their meals	403	27.8%
	Reduction of leftovers	265	18.3%
	Increase in conversation	468	32.3%
	Recognition of importance of oral care management	882	60.8%
	Implementation of independent self-oral care	808	55.7%
	Shortened hospital stay	143	9.9%
Medical team participation	Nutritional support team	81	5.6%
	Swallowing function support team	121	8.3%
	Oral care support team	199	13.7%
	Cancer care team	57	3.9%
	Palliative care team	57	3.9%
	Ventilation support team	18	1.2%
	Decubitus prevention team	12	0.8%
	Infection control team	53	3.7%
	Medical safety team	72	5.0%

Percentages were calculated as the number of dental hygienists implementing perioperative oral care management. The items in Table 1 were all dichotomous variables and multiple choice.

## Data Availability

Data is available for corresponding author by reasonable request.

## Supplementary Materials

The following are available online at https://www.mdpi.com/1660-4601/18/1/114/s1, Table S1: Three-parameter logistic model based on the item response theory, Table S2: Logistic regression analysis for the self-assessed effect of perioperative oral care management by practice of dental hygienist, Table S3: Cross-tabulations of the practice of perioperative oral care management against the self-assessed effect and participation in a medical team.
