# Supplementary material for: Dental Hygienists’ Practice in Perioperative Oral Care Management According to the Japanese Dental Hygienists Survey 2019
Source: Int J Environ Res Public Health. 2020 Dec 26;18(1):114. doi: 10.3390/ijerph18010114 (PMC7795937; doi:10.3390/ijerph18010114)
Supplement: Supplementary file 1 [file ijerph-18-00114-s001.pdf]

Supplemental materials
Dental hygienists' practice in perioperative oral care managements

From Japanese dental hygienists survey 2019

Table S1. Three parameter logistic model based on item response theory.

(A) Model 1. Practice of perioperative oral care management.

	Discrimination	Difficulty	Guessing
Cleaning of tongue and mucosa	2.62	−2.00	<0.01
Moisture retention if oral cavity	1.51	−3.10	<0.01
Topical fluoride application	2.85	−1.01	<0.01
Mouth rinsing instructions and gargling	3.05	−0.47	<0.01
Drug application for gingiva and oral mucosa	1.33	−0.82	<0.01
Oral health instruction for patients, their family or caregivers	2.18	−0.69	<0.01
Denture cleaning	1.49	−0.32	<0.01
Training of swallowing function	2.04	−1.85	<0.01
Assessment of oral conditions	2.14	−2.04	<0.01
Nutritional instruction	23.99	0.39	0.05
Cleaning of tongue and mucosa	10.58	0.33	0.12
Moisture retention if oral cavity	3.01	0.85	0.02

(B) Model 2. Self-assessed effects of perioperative oral care management.

	Discrimination	Difficulty	Guessing
Improvement of symptoms of tongue or oral mucosa	2.52	−0.55	<0.01
Improvement of symptoms of teeth or gingiva	2.80	−1.11	<0.01
Improvement of symptoms of oral hygiene status	3.47	−1.56	<0.01
Improvement of symptoms of xerostomia, improvement in salivary flow	2.53	−0.01	<0.01
Improvement of symptoms of malodor	2.43	−0.60	<0.01
Reduction of medication	2.79	1.47	0.01
Improvement of sensation of taste and patients enjoying their meals	3.51	0.49	0.01

Reduction of leftovers	4.03	0.83	0.01
Increase in conversation	3.93	0.38	0.04
Recognition of importance of oral care management	2.22	−0.67	0.05
Implementation of independent self-oral care	1.56	−0.59	<0.01
Shortened hospital stay	2.18	1.45	<0.01

(C) Model 3. Participation in medical care team.

	**Discrimination**	**Difficulty**	**Guessing**
Nutritional support team	36.65	1.04	0.01
Swallowing function support team	95.47	0.77	0.01
Oral care support team	47.22	0.55	0.07
Cancer care team	24.08	1.10	0.02
Palliative care team	17.20	1.10	0.01
Ventilation support team	3.45	1.73	<0.01
Decubitus prevention team	3.04	2.02	<0.01
Infection control team	16.34	1.14	0.02
Medical safety team	32.88	1.08	0.03

Table S2. Logistic regression analysis for self-assessed effect of perioperative oral care management by practice of dental hygienist.

		Multivariate Adjusted Odds Ratio (95% CI)	*p*-Value	Fitness	Odds ratio Stepwise Selection	*p*-Value	Fitness
Improvement of symptoms of tongue or oral mucosa	Tooth brushing by dental hygienist	1.248 (0.623–2.498)	0.532	0.236			0.692
	Scaling and mechanical tooth cleaning	1.711 (0.615–4.761)	0.304				
	Cleaning of tongue and mucosa	1.717 (1.158–2.546)	0.007		2.039(1.436–2.896)	<0.001	
	Moisture retention if oral cavity	1.348 (0.950–1.912)	0.095				
	Topical fluoride application	0.714 (0.517–0.985)	0.040				
	Mouth rinsing instructions and gargling	1.415 (1.012–1.979)	0.042		1.453(1.056–2.001)	0.022	
	Drug application for gingiva and oral mucosa	2.141 (1.594–2.876)	<0.001		2.047(1.542–2.717)	0.000	
	Oral health instruction for patients, their family or caregivers	2.078 (1.169–3.694)	0.013		2.086(1.222–3.562)	0.007	
	Denture cleaning	0.874 (0.450–1.696)	0.691				
	Training of swallowing function	1.886 (1.251–2.842)	0.002		2.101(1.430–3.087)	<0.001	
	Assessment of oral conditions	1.243 (0.888–1.740)	0.205				
	Nutritional instruction	1.894 (1.204-2.980)	0.006		1.988(1.276–3.098)	0.002	
Improvement of symptoms of teeth or gingiva	Tooth brushing by dental hygienist	1.518 (0.761–3.028)	0.236	0.331	2.134(1.152–3.954)	0.016	0.894
	Scaling and mechanical tooth cleaning	2.701 (1.013–7.199)	0.047				
	Cleaning of tongue and mucosa	1.150(0.734–1.801)	0.542				
	Moisture retention if oral cavity	1.122 (0.704-1.701)	0.588				
	Topical fluoride application	0.919 (0.639–1.322)	0.650				
	Mouth rinsing instructions and gargling	1.208 (0.819–1.780)	0.341				
	Drug application for gingiva and oral mucosa	2.305 (1.614–3.291)	<0.001		2.595(1.866–3.609)	0.000	
	Oral health instruction for patients, their family or caregivers	1.808 (1.030–3.175)	0.039		2.297(1.380–3.824)	0.001	
	Denture cleaning	1.163 (0.589–2.295)	0.663				
	Training of swallowing function	1.367 (0.837–2.233)	0.212				
	Assessment of oral conditions	1.056 (0.707–1.577)	0.789				
	Nutritional instruction	1.853 (1.051-3.264)	0.033		2.394(1.436–3.992)	0.001	

Improvement of symptoms of oral hygiene status	Tooth brushing by dental hygienist	1.252 (0.531–2.952)	0.607	0.102			0.702
	Scaling and mechanical tooth cleaning	6.170 (2.25–16.918)	<0.001		7.705(2.977-19.940)	<0.001	
	Cleaning of tongue and mucosa	0.684 (0.371–1.261)	0.224				
	Moisture retention if oral cavity	1.084 (0.621-1.891)	0.777				
	Topical fluoride application	1.186 (0.727–1.935)	0.495				
	Mouth rinsing instructions and gargling	1.749 (1.041–2.936)	0.035		1.954(1.230–3.103)	0.005	
	Drug application for gingiva and oral mucosa	1.269 (0.779–2.066)	0.339				
	Oral health instruction for patients, their family or caregivers	2.332 (1.210–4.496)	0.011		2.800(1.531–5.119)	0.001	
	Denture cleaning	1.308 (0.580–2.951)	0.517				
	Training of swallowing function	1.948 (0.917–4.140)	0.083		2.804(1.452–5.418)	0.002	
	Assessment of oral conditions	1.347 (0.759–2.393)	0.309				
	Nutritional instruction	1.499 (0.645–3.482)	0.347				
Improvement of symptoms of xerostomia, improvement in salivary flow	Tooth brushing by dental hygienist	0.903 (0.413–1.973)	0.798	0.969			0.377
	Scaling and mechanical tooth cleaning	1.798 (0.628–5.149)	0.274				
	Cleaning of tongue and mucosa	1.588 (1.026–2.459)	0.038		1.630(1.063–2.501)	0.025	
	Moisture retention if oral cavity	1.834 (1.295–2.596)	0.001		1.939(1.387–2.712)	<0.001	
	Topical fluoride application	0.959 (0.701–1.310)	0.791				
	Mouth rinsing instructions and gargling	2.019(1.432–2.848)	<0.001		2.017(1.449–2.808)	<0.001	
	Drug application for gingiva and oral mucosa	1.362 (1.021–1.816)	0.036		1.395(1.056–1.842)	0.019	
	Oral health instruction for patients, their family or caregivers	0.948(0.510–1.764)	0.866				
	Denture cleaning	1.372 (0.658–2.862)	0.399		1.720(1.243–2.378)	0.001	
	Training of swallowing function	1.584 (1.124–2.233)	0.009				
	Assessment of oral conditions	1.316 (0.966–1.795)	0.082				
	Nutritional instruction	2.068 (1.435–2.980)	<0.001		2.224(1.557–3.178)	<0.001	
Improvement of symptoms of malodor	Tooth brushing by dental hygienist	0.767 (0.399–1.476)	0.427	0.507			0.830
	Scaling and mechanical tooth cleaning	1.440 (0.533–3.890)	0.472				
	Cleaning of tongue and mucosa	1.518 (1.030–2.239)	0.035		1.604(1.130–2.278)	0.008	
	Moisture retention if oral cavity	1.202 (0.848–1.705)	0.301				
	Topical fluoride application	1.212(0.891-1.648)	0.222				

	Mouth rinsing instructions and gargling	1.667 (1.202–2.311)	0.002		1.802(1.323–2.455)	<0.001	
	Drug application for gingiva and oral mucosa	1.652 (1.232–2.213)	0.001		1.748(1.314–2.324)	<0.001	
	Oral health instruction for patients, their family or caregivers	1.107 (0.637–1.921)	0.719				
	Denture cleaning	1.945 (1.002–3.774)	0.049		2.117(1.154–3.886)	0.015	
	Training of swallowing function	1.605 (1.072–2.402)	0.021		1.611(1.081–2.401)	0.019	
	Assessment of oral conditions	1.328 (0.949–1.857)	0.098		1.390(1.006–1.920)	0.046	
	Nutritional instruction	1.851 (1.179–2.904)	0.007		1.896(1.210–2.969)	0.005	
	Tooth brushing by dental hygienist	0.673 (0.161–2.805)	0.587				
	Scaling and mechanical tooth cleaning	0.503 (0.092–2.763)	0.429				
	Cleaning of tongue and mucosa	0.497 (0.213–1.158)	0.105				
	Moisture retention if oral cavity	1.270 (0.606–2.662)	0.527				
	Topical fluoride application	3.222 (1.529–6.790)	0.002		3.128(1.571–6.231)	0.001	
	Mouth rinsing instructions and gargling	2.062 (0.929–4.578)	0.075				
Reduction of medication	Drug application for gingiva and oral mucosa	1.814 (1.022–3.219)	0.042	0.408	1.956(1.134–3.375)	0.016	0.635
	Oral health instruction for patients, their family or caregivers	0.806 (0.207–3.133)	0.755				
	Denture cleaning	0.698 (0.172–2.838)	0.616		2.068(1.286–3.325)	0.003	
	Training of swallowing function	2.062 (1.199–3.544)	0.009				
	Assessment of oral conditions	0.993 (0.562–1.754)	0.981				
	Nutritional instruction	2.707(1.661–4.411)	<0.001		2.695(1.692–4.293)	<0.001	
	Tooth brushing by dental hygienist	0.606 (0.274–1.343)	0.217				
	Scaling and mechanical tooth cleaning	0.822 (0.279–2.427)	0.723				
	Cleaning of tongue and mucosa	0.764 (0.470–1.241)	0.276				
	Moisture retention if oral cavity	1.279 (0.848–1.929)	0.241				
Improvement of sensation of taste and patients enjoying their meals	Topical fluoride application	1.425 (1.001–2.028)	0.049	0.760	1.438(1.023–2.023)	0.037	0.364
	Mouth rinsing instructions and gargling	1.511 (1.011–2.259)	0.044		1.441(0.995–2.087)	0.053	
	Drug application for gingiva and oral mucosa	1.622 (1.176–2.237)	0.003		1.659(1.207–2.281)	0.002	
	Oral health instruction for patients, their family or caregivers	0.911 (0.444–1.871)	0.800				

	Denture cleaning	1.740 (0.694–4.363)	0.237				
	Training of swallowing function	1.381 (0.974–1.958)	0.07		1.414(1.002–1.994)	0.049	
	Assessment of oral conditions	1.592 (1.137–2.230)	0.007		1.614(1.168–2.230)	0.004	
	Nutritional instruction	2.699 (1.912–3.809)	<0.001		2.673(1.896–3.768)	<0.001	
	Tooth brushing by dental hygienist	0.334 (0.132–0.849)	0.021		0.384(0.162–0.907)	0.029	
	Scaling and mechanical tooth cleaning	1.364 (0.343–5.417)	0.659				
	Cleaning of tongue and mucosa	0.777 (0.407–1.483)	0.444				
	Moisture retention if oral cavity	1.599 (0.932–2.741)	0.088				
	Topical fluoride application	1.449 (0.935–2.246)	0.097		1.564(1.020–2.396)	0.040	
	Mouth rinsing instructions and gargling	1.643 (0.974–2.772)	0.063		1.775(1.078–2.922)	0.024	
Reduction of leftovers	Drug application for gingiva and oral mucosa	1.416 (0.955–2.100)	0.084	0.576	1.447(0.978–2.142)	0.064	0.004
	Oral health instruction for patients, their family or caregivers	1.249 (0.443–3.519)	0.674				
	Denture cleaning	1.148 (0.388–3.402)	0.803				
	Training of swallowing function	1.467 (0.989–2.175)	0.056		1.513(1.025–2.233)	0.037	
	Assessment of oral conditions	1.929 (1.286–2.894)	0.001		2.078(1.403–3.077)	0.000	
	Nutritional instruction	2.791 (1.922–4.055)	<0.001		2.784(1.920–4.037)	<0.001	
	Tooth brushing by dental hygienist	0.674 (0.300–1.515)	0.34				
	Scaling and mechanical tooth cleaning	0.943 (0.306–2.900)	0.918				
	Cleaning of tongue and mucosa	1.139 (0.715–1.814)	0.583				
	Moisture retention if oral cavity	1.062 (0.726–1.553)	0.757				
	Topical fluoride application	1.381 (0.992–1.924)	0.056		1.731(1.228–2.441)	0.002	
	Mouth rinsing instructions and gargling	1.561 (1.073–2.269)	0.020		1.510(1.123–2.030)	0.006	
Increase in conversation	Drug application for gingiva and oral mucosa	1.400 (1.033–1.897)	0.030	0.759			0.632
	Oral health instruction for patients, their family or caregivers	1.256 (0.595–2.652)	0.550				
	Denture cleaning	3.739 (1.246–11.223)	0.019		4.044(1.415–11.558)	0.009	
	Training of swallowing function	1.542 (1.099–2.162)	0.012		1.559(1.117–2.176)	0.009	
	Assessment of oral conditions	1.805 (1.312–2.484)	<0.001		1.882(1.386–2.556)	<0.001	
	Nutritional instruction	2.126 (1.504–3.005)	<0.001		2.178(1.544–3.072)	<0.001	
Recognition of importance of oral care management	Tooth brushing by dental hygienist	0.912 (0.461–1.804)	0.791	0.280			0.683

	Scaling and mechanical tooth cleaning	2.104 (0.736–6.019)	0.165				
	Cleaning of tongue and mucosa	1.156 (0.775-1.722)	0.478				
	Moisture retention if oral cavity	0.951 (0.660–1.368)	0.785				
	Topical fluoride application	1.318 (0.962–1.805)	0.085		1.433(1.060–1.936)	0.019	
	Mouth rinsing instructions and gargling	2.006 (1.434–2.808)	<0.001		2.252(1.655–3.064)	0.000	
	Drug application for gingiva and oral mucosa	1.317 (0.970–1.787)	0.077				
	Oral health instruction for patients, their family or caregivers	1.655 (0.941–2.912)	0.081		1.781(1.017–3.120)	0.044	
	Denture cleaning	2.636 (1.337–5.198)	0.005		3.094(1.604–5.967)	0.001	
	Training of swallowing function	1.452 (0.950–2.219)	0.084				
	Assessment of oral conditions	1.870 (1.315–2.660)	<0.001		2.070(1.503–2.850)	<0.001	
	Nutritional instruction	1.942(1.195–3.156)	0.007		2.318(1.458–3.684)	<0.001	
	Tooth brushing by dental hygienist	1.809 (0.912–3.588)	0.09				
	Scaling and mechanical tooth cleaning	1.571 (0.570–4.326)	0.383				
	Cleaning of tongue and mucosa	1.154 (0.780–1.706)	0.473				
	Moisture retention if oral cavity	0.816 (0.576–1.157)	0.254				
	Topical fluoride application	1.186 (0.879–1.600)	0.264				
	Mouth rinsing instructions and gargling	1.421 (1.024–1.972)	0.035		1.563(1.171–2.085)	0.002	
Implementation of independent self-oral care	Drug application for gingiva and oral mucosa	1.232 (0.925–1.641)	0.153	0.318			
	Oral health instruction for patients, their family or caregivers	2.237 (1.266–3.951)	0.006		2.467(1.409–4.320)	0.002	
	Denture cleaning	2.243 (1.141–4.411)	0.019		2.730(1.434–5.199)	0.002	
	Training of swallowing function	0.750 (0.521–1.080)	0.122				
	Assessment of oral conditions	1.839 (1.328–2.548)	<0.001		1.653(1.240–2.204)	0.001	
	Nutritional instruction	1.838 (1.243–2.719)	0.002		1.750(1.214–2.521)	0.003	
	Tooth brushing by dental hygienist	0.800 (0.160–3.998)	0.786				
	Scaling and mechanical tooth cleaning	0.705 (0.138–3.593)	0.674				
	Cleaning of tongue and mucosa	1.158 (0.454–2.951)	0.759				
Shortened hospital stay	Moisture retention if oral cavity	1.684 (0.826–3.431)	0.151	0.038	2.357(1.284–4.327)	0.006	0.554
	Topical fluoride application	1.824 (0.994–3.347)	0.052		1.896(1.062–3.385)	0.031	
	Mouth rinsing instructions and gargling	1.840 (0.888–3.812)	0.101				

	Drug application for gingiva and oral mucosa	1.653 (0.976–2.799)	0.062	1.931(1.163–3.204)	0.011
	Oral health instruction for patients, their family or caregivers	0.820 (0.225–2.992)	0.764		
	Denture cleaning	1.127 (0.228–5.578)	0.883		
	Training of swallowing function	1.302 (0.789–2.147)	0.301		
	Assessment of oral conditions	1.234 (0.734–2.074)	0.427		
	Nutritional instruction	2.348 (1.472–3.745)	<0.001	2.999(2.016–4.460)	<0.001

Multivariate Odds ratios and odds ratios by stepwise selection were calculated. Fitness indexes were calculated by Hosmer-Lemeshow test.

Table S3. Cross tabulations of practice of perioperative oral care management against self-assessed effect and participation in medical team.

		Tooth Brushing by Dental Hygienist			Scaling and Mechanical Tooth Cleaning			Cleaning off TONGUE and Mucosa			Moisture Retention if Oral Cavity		
		−	+	*p*-Value	−	+	*p*-Value	−	+	*p*-Value	−	+	*p*-Value
Improvement of symptoms of oral mucosa or tongue	-	34	370	<0.001	14	390	0.012	133	269	<0.001	208	192	<0.001
	+	18	816		11	822		84	748		188	642	
Improvement of symptoms of tooth or gingiva	-	23	195	<0.001	12	206	<0.001	66	152	<0.001	107	109	<0.001
	+	30	1003		13	1020		151	879		294	732	
Improvement of symptoms of oral hygiene status	-	14	93	<0.001	13	94	<0.001	32	75	0.001	55	50	<0.001
	+	41	1124		13	1152		195	966		356	800	
Improvement of symptoms of xerostomia, improvement salivary flow	-	42	581	<0.001	18	605	0.025	175	446	<0.001	300	318	<0.001
	+	11	615		7	619		43	581		99	524	
Improvement of symptoms of malodor	-	31	360	<0.001	14	377	0.007	125	265	<0.001	198	189	<0.001
	+	24	835		11	848		94	763		202	653	
Reduction in medication	-	49	1048	0.254	23	1074	0.678	205	889	0.008	379	712	<0.001
	+	3	126		2	127		12	117		17	112	
Improvement of sensation of taste and enjoy their meal	-	42	783	0.056	19	806	0.343	176	646	<0.001	324	495	<0.001
	+	11	392		6	397		40	363		70	331	
Reduction of leftovers	-	44	903	0.248	23	924	0.198	196	748	<0.001	359	582	<0.001
	+	8	257		3	262		20	245		33	232	
Increase of conversation	-	43	707	0.003	19	731	0.134	175	573	<0.001	309	436	<0.001
	+	10	458		6	462		39	428		83	384	
Recognition of importance of oral care management	-	31	323	<0.001	16	338	<0.001	110	244	<0.001	177	175	<0.001
	+	22	860		9	873		108	771		220	657	
Implementation of Independent self oral care	-	37	393	<0.001	16	414	0.002	110	319	<0.001	180	247	<0.001
	+	16	792		9	799		107	699		218	584	
Shortening hospital stay	-	49	991	0.067	24	1016	0.487	201	837	<0.001	369	666	<0.001
	+	2	141		2	141		7	135		15	127	

		Fluoride Varnish			Instruction of Mouth Rinse and Gargling			Drug Application for Gingiva and Oral Mucosa			Oral Health Instruction for Patients, Their Family or Caregivers		
		−	+	*p*-Value	−	+	*p*-Value	−	+	*p*-Value	−	+	*p*-Value
Improvement of symptoms of oral mucosa or tongue	-	158	243	<0.001	183	219	<0.001	243	160	<0.001	56	348	<0.001
	+	213	614		154	677		247	582		25	806	
Improvement of symptoms of tooth or gingiva	-	96	121	<0.001	101	117	<0.001	142	76	<0.001	36	182	<0.001
	+	280	744		243	784		354	673		45	985	
Improvement of symptoms of oral hygiene status	-	57	50	<0.001	59	48	<0.001	66	41	<0.001	26	81	<0.001
	+	328	825		291	865		439	717		56	1104	
Improvement of symptoms of xerostomia, improvement salivary flow	-	241	378	<0.001	261	360	<0.001	325	297	<0.001	63	559	<0.001
	+	134	486		82	541		169	452		19	604	
Improvement of symptoms of malodor	-	173	217	<0.001	183	207	<0.001	233	157	<0.001	50	340	<0.001
	+	201	651		162	693		264	590		32	825	
Reduction in medication	-	362	728	<0.001	330	764	<0.001	471	623	<0.001	79	1015	<0.001
	+	10	119		10	119		19	110		3	126	
Improvement of sensation of taste and enjoy their meal	-	301	518	<0.001	283	539	<0.001	398	424	<0.001	70	753	<0.001
	+	70	329		55	346		90	312		12	389	
Reduction of leftovers	-	329	613	<0.001	308	636	<0.001	433	511	<0.001	77	867	<0.001
	+	40	223		28	237		53	212		5	260	
Increase of conversation	-	285	462	<0.001	272	477	<0.001	372	377	<0.001	71	678	<0.001
	+	85	379		63	404		114	351		10	455	
Recognition of importance of oral care management	-	165	188	<0.001	174	180	<0.001	205	148	<0.001	55	298	<0.001
	+	209	665		167	711		284	592		26	853	
Implementation of Independent self oral care	-	176	254	<0.001	172	257	<0.001	222	207	<0.001	58	370	<0.001
	+	197	599		167	636		269	532		23	783	
Shortening hospital stay	-	351	684	<0.001	320	718	<0.001	453	586	<0.001	78	960	0.017
	+	16	126		11	131		24	118		3	139	

		Denture Cleaning			Training of Swallowing Function			Assessment of Oral Conditions			Nutritional Instruction		
		−	+	*p*-Value	−	+	*p*-Value	−	+	*p*-Value	−	+	*p*-Value
Improvement of symptoms of oral mucosa or tongue	-	35	368	<0.001	348	55	<0.001	292	109	<0.001	363	35	<0.001
	+	24	808		478	346		365	458		558	256	
Improvement of symptoms of tooth or gingiva	-	24	194	<0.001	182	36	<0.001	150	66	<0.001	196	20	<0.001
	+	34	995		653	367		513	508		735	273	
Improvement of symptoms of oral hygiene status	-	17	90	<0.001	94	13	<0.001	80	26	<0.001	98	8	<0.001
	+	44	1117		754	394		593	555		846	288	
Improvement of symptoms of xerostomia, improvement salivary flow	-	46	575	<0.001	512	108	<0.001	426	192	<0.001	549	66	<0.001
	+	13	612		321	296		234	383		378	230	
Improvement of symptoms of malodor	-	38	351	<0.001	333	56	<0.001	280	108	<0.001	353	33	<0.001
	+	20	837		502	346		381	467		577	261	
Reduction in medication	-	56	1038	0.161	781	308	<0.001	621	467	<0.001	867	212	<0.001
	+	3	126		45	84		36	93		53	74	
Improvement of sensation of taste and enjoy their meal	-	51	772	0.001	635	185	<0.001	523	294	<0.001	703	109	<0.001
	+	7	395		189	208		130	268		213	179	
Reduction of leftovers	-	54	891	0.011	715	227	<0.001	584	355	<0.001	791	144	<0.001
	+	5	259		104	158		66	198		120	138	
Increase of conversation	-	53	695	<0.001	595	152	<0.001	496	248	<0.001	646	94	<0.001
	+	4	462		224	238		154	310		264	191	
Recognition of importance of oral care management	-	41	312	<0.001	304	49	<0.001	267	85	<0.001	323	27	<0.001
	+	17	863		523	348		388	482		596	265	
Implementation of Independent self oral care	-	42	388	<0.001	328	101	<0.001	293	134	<0.001	370	54	<0.001
	+	17	788		502	294		364	434		550	237	
Shortening hospital stay	-	56	981	0.039	746	289	<0.001	598	434	<0.001	829	197	<0.001
	+	2	140		56	84		38	104		64	73	

		Tooth Brushing by Dental Hygienist			Scaling and Mechanical Tooth Cleaning			Cleaning off Tongue and Mucosa			Moisture Retention if Oral Cavity		
		−	+	*p*-Value	−	+	*p*-Value	−	+	*p*-Value	−	+	*p*-Value
Nutritional support team	-	72	1234	0.094	35	1269	0.907	265	1033	<0.001	474	818	<0.001
	+	1	80		2	79		2	79		2	79	
Swallowing function support team	-	71	1196	0.063	33	1232	0.649	265	994	<0.001	470	783	<0.001
	+	2	119		4	117		2	119		6	115	
Oral care support team	-	68	1122	0.061	33	1155	0.534	259	923	<0.001	457	720	<0.001
	+	5	194		4	195		8	191		19	179	
Cancer care team	-	72	1255	0.225	37	1288	0.201	266	1053	0.001	471	842	<0.001
	+	1	56		0	57		1	56		5	52	
palliative care team	-	72	1255	0.225	35	1290	0.691	267	1052	<0.001	474	839	<0.001
	+	1	56		2	55		0	57		2	55	
Ventilation support team	-	73	1291	0.313	36	1326	0.447	267	1089	0.036	475	875	0.009
	+	0	18		1	17		0	18		1	17	
Decubitus prevention team	-	73	1297	0.411	37	1331	0.564	267	1095	0.087	475	881	0.053
	+	0	12		0	12		0	12		1	11	
Infection control team	-	72	1260	0.261	36	1294	0.717	264	1060	0.010	465	853	0.029
	+	1	52		1	52		3	50		11	42	
Medical safety team	-	72	1235	0.129	36	1269	0.484	262	1037	0.006	462	831	0.001
	+	1	71		1	71		5	67		12	60	

		Fluoride Varnish			Instruction of Mouth Rinse and Gargling			Drug Application for Gingiva and Oral Mucosa			Oral Health Instruction for Patients, Their Family or Caregivers		
		−	+	*p*-Value	−	+	*p*-Value	−	+	*p*-Value	−	+	*p*-Value
Nutritional support team	-	419	873	<0.001	404	890	0.000	568	724	<0.001	108	1192	0.022
	+	10	71		6	75		15	65		1	80	
Swallowing function support team	-	410	843	<0.001	398	857	0.000	556	697	<0.001	105	1156	0.050
	+	19	102		12	109		27	93		4	117	
Oral care support team	-	386	790	0.002	385	794	0.000	530	647	<0.001	101	1083	0.029
	+	43	156		25	173		54	144		8	191	
Cancer care team	-	421	892	0.004	407	908	0.000	574	739	<0.001	109	1212	0.024
	+	8	49		3	54		9	47		0	57	
palliative care team	-	421	892	0.004	407	908	0.000	574	739	<0.001	107	1214	0.209
	+	8	49		3	54		9	48		2	55	
Ventilation support team	-	426	924	0.063	410	942	0.005	579	771	0.078	108	1250	0.708
	+	2	16		0	18		4	14		1	17	
Decubitus prevention team	-	427	929	0.085	409	949	0.101	581	775	0.068	108	1256	0.958
	+	1	11		1	11		2	10		1	11	
Infection control team	-	421	897	0.004	403	917	0.007	571	747	0.007	106	1220	0.537
	+	7	46		7	46		13	40		3	50	
Medical safety team	-	419	874	0.000	397	898	0.012	565	728	0.001	104	1197	0.454
	+	8	64		12	60		17	55		4	68	

		Denture Cleaning			Training of Swallowing Function			Assessment of Oral Conditions			Nutritional Instruction		
		−	+	*p*-Value	−	+	*p*-Value	−	+	*p*-Value	−	+	*p*-Value
Nutritional support team	-	84	1217	0.018	924	365	<0.001	753	533	<0.001	1018	256	<0.001
	+	0	81		21	60		9	71		29	47	
Swallowing function support team	-	82	1180	0.033	916	334	<0.001	744	503	<0.001	998	237	<0.001
	+	2	119		29	92		18	102		49	67	
Oral care support team	-	77	1108	0.103	866	308	<0.001	709	462	<0.001	943	218	<0.001
	+	7	192		78	120		52	145		107	86	
Cancer care team	-	84	1238	0.050	924	386	<0.001	747	560	<0.001	1024	270	<0.001
	+	0	57		20	37		14	42		23	31	
palliative care team	-	82	1240	0.405	928	382	<0.001	751	556	<0.001	1027	266	<0.001
	+	2	55		16	41		10	46		20	36	
Ventilation support team	-	84	1275	0.276	939	408	<0.001	757	587	0.004	1043	287	<0.001
	+	0	18		4	14		4	14		5	13	
Decubitus prevention team	-	84	1281	0.375	941	412	<0.001	760	590	<0.001	1046	290	<0.001
	+	0	12		2	10		1	11		2	10	
Infection control team	-	83	1244	0.192	920	395	<0.001	744	568	0.003	1023	276	<0.001
	+	1	52		25	28		19	34		25	26	
Medical safety team	-	82	1220	0.225	909	381	<0.001	738	549	<0.001	1009	265	<0.001
	+	2	70		33	39		22	50		36	34	
